# Photometric light curve analysis of three overcontact binary systems: ATO J255.8159+16.8821, CRTS J034336.4+264312, and NSVS 2669503

**DOI:** 10.1038/s41598-025-12402-y

**Published:** 2025-08-04

**Authors:** Ahmed Shokry, Mohamed S. Darwish, Mohamed A. El-Sadek, Gamal M. Hamed, Ibrahim Zead, Ali Takey

**Affiliations:** Astronomy Department, National Research Institute of Astronomy and Geophysics (NRIAG), Helwan, Cairo, 11421 Egypt

**Keywords:** Binaries, Eclipsing stars, Fundamental parameters, Stars: individual (ATO J255.8159+16.8821, CRTS J034336.4+264312, NSVS 2669503), Astronomy and astrophysics, Stars, Stellar evolution

## Abstract

Multi-band photometric observations of three contact binaries (ATO J255.8159+16.8821, CRTS J034336.4+264312, and NSVS 2669503) were carried out using the 1.88 m telescope at the Kottamia Astronomical Observatory (KAO) in Egypt. New times of minima for all three systems have been calculated. In particular, for NSVS 2669503, we performed an $O-C$ analysis to investigate period variations. The results indicate a decreasing orbital period, with a rate of $\textrm{d}P/\textrm{d}t \approx 1.85 \times 10^{-10}$ days yr$\phantom{0}^{-1}$. Analysis using the Wilson-Devinney (W-D) program revealed that all three systems are A-subtype contact binaries with mass ratios (q) of 0.47, 0.34, and 0.28, respectively. The results showed that ATO J255.8159+16.8821 exhibits the O’Connell effect, while the other two systems (CRTS J034336.4+264312 and NSVS 2669503) have a symmetric light curve. The fill-out factors of the systems were determined to be 0.204, 0.157, and 0.069, respectively, indicating all three systems are shallow contact systems. To understand their evolutionary status, mass-luminosity and mass-radius diagrams were plotted. These diagrams indicate that the primary components of the three systems are main sequence stars, whereas the less massive stars have evolved beyond the main sequence. The dynamical evolution of the systems is also discussed.

## Introduction

Short period contact binary systems of the W Ursae Majoris (W UMa) type represent a significant fraction of eclipsing binaries observed in the Galaxy^1,2^. These systems typically consist of two late-type stars^3,4^ Both stars fill or even exceed their Roche lobes^5^. Occasionally, contact binaries are observed in the early stages of their interaction, known as the shallow contact or pre-contact phase. Shallow contact systems are those systems that have a fill-out ratio $f < 25\%$. This phase provides a valuable opportunity to investigate the evolutionary status of such systems^6–8^. It is typically characterized by components with differing effective temperatures, leading to a light curve that exhibits unequal eclipse depths for the primary and secondary minima. Despite its significance, this phase remains relatively rare among observed systems. Further details on this type of binary and its evolutionary path can be found in^9^. Contact binary systems are classified into two subtypes based on their spectral characteristics and physical properties: the A-subtype and the W-subtype, as initially proposed by^10^. For A-subtype binaries, the more massive component is hotter than the less massive one, and the mass ratio (M2/M1) is generally less than 0.5, while W-subtype systems are distinguished by having the less massive component as the hotter star, and they often show continuous period changes over time.^11,12^. Contact binary systems with low mass ratios are believed to be potential progenitors of unusual stellar types, including FK Comae stars and blue stragglers^13,14^. If the mass ratio decreases below a critical theoretical limit ($q_{\textrm{min}}$), the system may undergo Darwin instability^15,16^, resulting in the merging of binary components into a single star.^17^ found that this threshold can be as low as $q = 0.044$. Conversely,^18^ reported that the mass ratio in contact binaries can reach values as high as 0.72, a regime where the efficiency of energy transfer is lower compared to systems with the same luminosity ratio but different configurations. Furthermore, these systems are thought to be magnetically active, showing asymmetry in the shape of their light curves^19–21^. This phenomenon is usually known as the O’Connell effect^22^.

In the present study, we introduce photometric light curve analysis of three overcontact systems, $ATO J255.8159+16.8821$ (hereinafter ATO J255), $CRTS J034336.4+264312$ (hereinafter CRTS J034), and *NSVS*2669503 (hereinafter NSVS 266). The three systems were selected based on the criteria of systems having a short period (i.e., < 0.3 days) to better understand the physical parameters and evolutionary status of systems near the Contact binary period cut-off and their visibility at the Kottamia Astronomical Observatory (KAO) during our access time of the telescope. Of the three systems, CRTS J034 and ATO J255 are analyzed for the first time in this study. While *NSVS*2669503 is previously introduced by^23^, a new CCD photometric observation and more detailed analysis are reported in our study.

The system $ATO J255.8159+16.8821$ (also known as *2MASS J17031584+1652559* and *ZTF J170315.83+165255.5*) has been classified as an EW-type eclipsing binary by the Zwicky Transient Facility (ZTF;^24^), with a visual magnitude of $15^{\textrm{m}}.667$ and an orbital period of $0^{\textrm{d}}.2517454$. $CRTS J034336.4+264312$ (also identified as *ATO J055.9018+26.7202* and *ZTF J034336.43+264312.9*) was classified as an EW-type system by the Catalina Real-Time Transient Survey (CRTS;^25^), exhibiting a visual magnitude of $16^{\textrm{m}}.01$ and an orbital period of $0^{\textrm{d}}.252665$. On the other hand, *NSVS*2669503 (*2MASS J12501739+5231350*, *ZTF J125017.31+523134.5*) was classified as a W UMa-type (EW-type) contact binary by^26^. The system was also identified as an EW-type eclipsing binary by automated variable star classification in the Northern Sky Variability Survey (NSVS;^26^), with a visual magnitude of $13^{\textrm{m}}.69$ and an orbital period of $0^{\textrm{d}}.234003$. Photometric data for the system were presented in the Catalina Surveys Data Release 1 (CSS-DR1;^25^).^23^ performed a photometric study for the system and derived its orbital parameters.

The main aim of this work is to estimate the physical and absolute parameters of the three systems using new CCD photometric observations carried out by the 1.88-m telescopes at KAO as well as archival data. This analysis will help understand their evolution within the contact binary systems.

The paper is structured as follows. Section 2 describes the photometric observations and data reduction procedures. Section 3 presents the analysis of the period changes. Section 4 provides the results and discussion, including light curve solutions, calculations of absolute parameters, and the evolutionary status of the studied systems. Finally, Section 5 summarizes the findings and presents the conclusions.

## Observations and data reduction

### KAO observations

New photometric observations were carried out over several nights in 2024 for the three systems in the *V*, *R*, and *I* filters, using the Kottamia Faint Imaging Spectropolarimeter (KFISP) attached to the Cassegrain focus of the 1.88-m telescope at the Kottamia Astronomical Observatory (KAO)^27^. KAO observatory is located at an elevation of 476 meters above sea level, with coordinates of $29^\circ 55^\prime 35.24^{\prime \prime }$ N and $31^\circ 49^\prime 45.85^{\prime \prime }$ E. The observations were conducted using a $2\text {k} \times 2\text {k}$ CCD camera attached to the KFISP spectropolarimeter^27^. The CCD camera was cooled to an average operating temperature of $-120^\circ$C to minimize thermal noise. A binning mode $2 \times 2$ was applied to enhance the signal-to-noise ratio. The filter set used followed the standard Johnson-Cousin photometric system. A detailed log of the KAO observations, including dates, exposure times, and the number of frames, is presented in Table 1.

**Fig. 1:**
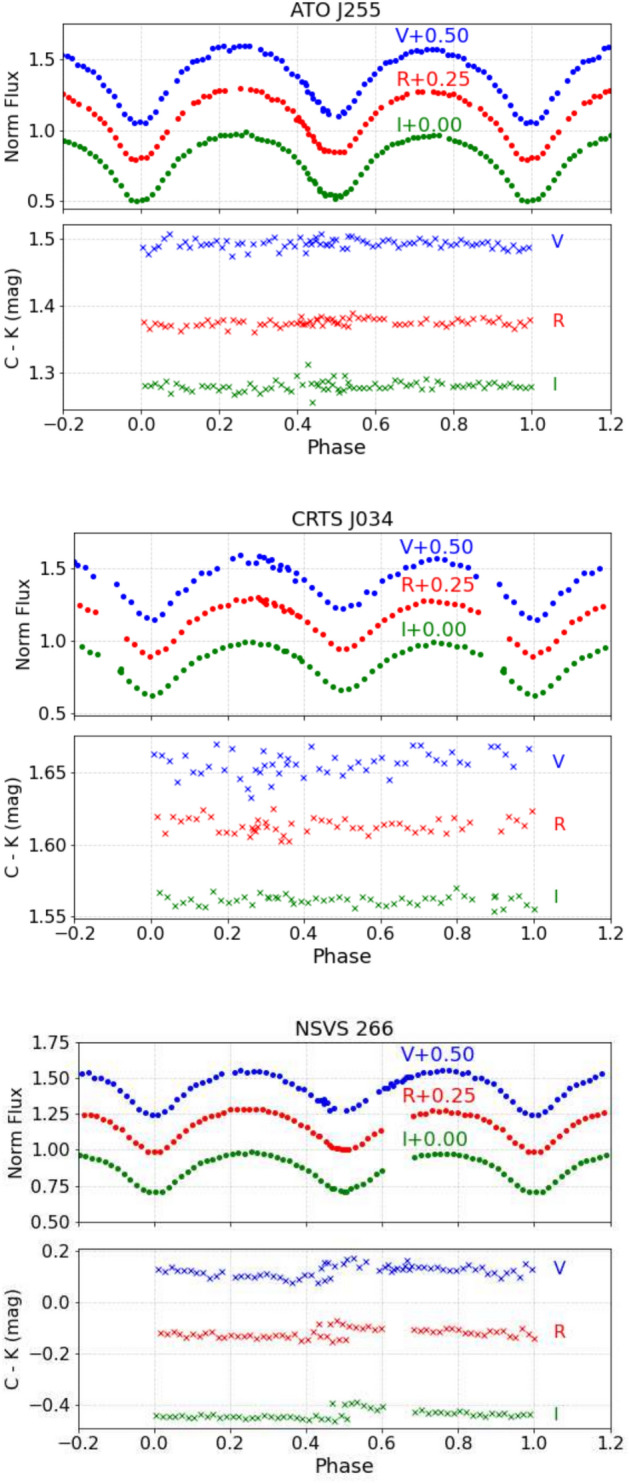
The observed light curves (Phase & normalized flux) of ATO J255 (top panel), CRTS J034 (middle panel), and NSVS 266 (bottom panel) in Johnson VRI filters. For each plot, the corresponding lower sub-panel shows the differential magnitude between the comparison and check stars.

Data reduction of raw CCD images was performed using the C-Munipack package, https://c-munipack.sourceforge.net/, where bias subtraction and flat-field correction were applied to the images following the standard method. C-Munipack provides a complete solution for the reduction of CCD images. It is particularly aimed at observations of variable stars. The magnitudes of the variable, comparison, and check stars in each frame were extracted using aperture photometry methods incorporating standard DAOPHOT procedures^28^. Differential photometry was performed with respect to comparison and check stars, and all times were corrected to HJD. Table 2 lists the journal of the variable, comparison, and check star for each system. Magnitudes were obtained from the Guide Star Catalogue, while coordinates were retrieved from Gaia DR3^29^. Figure 1 displays the measured light curves of the three systems in the Johnson V, R, and I filters, illustrated as normalized flux versus phase. The corresponding lower sub-panels in each plot display the differential magnitudes between the comparison and check stars, serving as a consistency check for the stability of the comparison star and the quality of the photometric observations.

**Table 1:** Observation Log for three targets. For each system, the filters used, the observation date, exposure time, and the number of observations in each filter.

System	Filter	Date	Exposure time (s)	No. observations
ATO J255	V, R, I	2024-07-24	150 – 110 – 100	80 – 73 – 82
CRTS J034	V, R, I	2024-01-03	180 – 120 – 120	53 – 55 – 53
NSVS 266	V, R, I	2024-03-19	120 – 80 – 80	67 – 61 – 59

**Table 2:** Variable comparison and check the stars’ coordinates of the three systems.

System	RA	Dec	V mag
ATO J255.8159+16.8821	17 03 15.83	+16 52 55.54	15.67
Comparison	17 03 29.05	+16 51 57.51	15.28
check	17 03 26.20	+16 54 32.36	14.00
CRTS J034336.4+264312	03 43 36.43	+26 43 13.01	16.15
Comparison	03 43 29.27	+26 44 57.55	14.82
check	03 43 37.20	+26 41 50.10	13.65
NSVS 2669503	12 50 17.40	+52 31 35.02	13.40
Comparison	12 50 33.71	+52 28 07.70	14.18
check	12 50 26.74	+52 33 22.08	14.31

### ZTF and TESS observations

Additional archival data were gathered to enhance the reliability of the light curve analysis.

For the three systems, we retrieved observational data from the Zwicky Transient Facility (ZTF) databases, which are available through the ZTF DR23 PublicReleases https://www.ztf.caltech.edu/ztf-public-releases.html. The ZTF observations were conducted using the g and r filters. The light curves were created using measurements from calibrated catalogs of single exposures derived by fitting the point spread function (PSF) to ensure no contamination from nearby systems^30^. More details on ZTF data reduction can be found in the study by^31^.

In addition to ZTF data we searched for available photometric observations of our systems in the Transiting Exoplanet Survey Satellite (TESS). TESS, a NASA mission aimed at detecting transiting exoplanets through an all-sky survey, provides both light curves and full-frame image cutouts, with photometric data processed into magnitudes^32^. We retrieved TESS data (HLSP mission) with an exposure time of 200 seconds for the NSVS 266 system from the MAST data archive (https://archive.stsci.edu/missions-and-data/tess). However, the available data for ATO J025 were insufficient to obtain a reliable fit, and no TESS data were available for CRTS J034.

## Eclipsing times and orbital period variation

For each system, we identified the primary and secondary times of minima for the V, R, and I filters using the method outlined by^33^, as presented in Table 3. The resultant light minimum times of the three systems are the averages of the light minimum times in the observed bands. The periods of the three systems were obtained from the literature. Based on our estimations of the times of light minima and the available periods, the new ephemerides for each system are given by the following equations:

1 $$\begin{aligned} ATO J255 : HJD (MinI) = 2460486.40346 (\pm 0.00074) + 0.251745^d \times E \end{aligned}$$

2 $$\begin{aligned} CRTS J034: HJD (MinI) = 2460313.3785 (\pm 0.00030) + 0.252665^d \times E \end{aligned}$$

For NSVS 266, we collected all the available data on light minimum times from the literature. There are 4 light minimum times of NSVS 266 reported in the literature by^23^, 8 from TESS data, and 2 minimum, including primary and secondary, observed by us. These minima are listed in Table 4, where the epoch, calculated from the ephemeris extracted from TESS data HJD(min) = 2453338.922367, and the period is 0.23400259$\phantom{0}^{d}$.

**Fig. 2:**
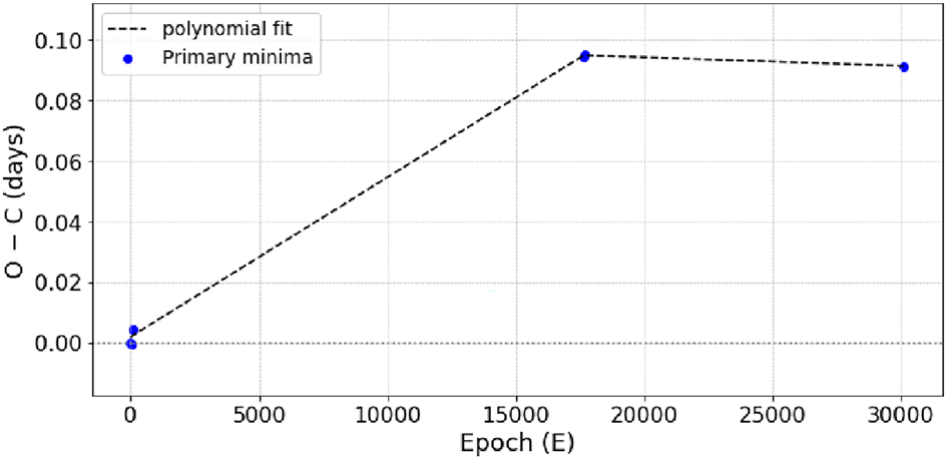
Variation plot of the (O–C)$\phantom{0}_{Primary}$ vs. Epoch relationship of NSVS 266. The blue circles are the photometrically derived O–C values, and the black dashed line is our polynomial fitting.

In Table 4, the parameter O–C is defined as the difference between the minimum time of observation and the calculation. In order to study the period variation, we plotted the (O–C)$\phantom{0}_{primary}$ versus the number of integer cycles (E) in Fig. 2, which shows an obvious upward parabolic trend. Therefore, we used a quadratic curve to fit this trend. The resultant quadratic ephemeris is:

3 $$\begin{aligned} NSVS 266 : HJD (MinI) = 2453338.922367 (\pm 0.000001) + 0.23400259^d(\pm 0.00000004) \times E+(-2.6619 \times 10^{-7})\times E^2 \end{aligned}$$

This ephemeris is used to calculate the phases and draw the light curves in the V, R, and I bands, as shown in Fig. 1. The quadratic term (Q) of this equation was found to be   − 2.6619 $\times$ 10$\phantom{0}^{-7}$, suggesting that the orbital period variation is decreasing by a rate of dP/dt   1.8485 $\times$ 10$\phantom{0}^{-10}$ days yr$\phantom{0}^{-1}$.

**Table 3:** Times of primary (*MinI*) and secondary minima (*MinII*) of the three systems.

Object	Filter	MinI (HJD)	MinII (HJD)
ATO J255	V	2460486.40330±0.00021	2460486.27830±0.00008
	R	2460486.40350±0.00026	2460486.27820±0.0002
	I	2460486.40360±0.00018	2460486.27820±0.00022
CRTS J034	V	2460313.3726±0.00053	2460313.24700±0.00059
	R	2460313.37190±0.00052	2460313.24720±0.00017
	I	2460313.37240±0.00018	2460313.24670±0.00024
NSVS 266	V	2460389.51200±0.00022	2460389.39510±0.00074
	R	2460389.51160± 0.00016	2460389.39530±0.00018
	I	2460389.51190±0.00014	2460389.39560±0.00023

**Table 4:** Times of minima and O–C values for NSVS 2669503 based on $T_0 = 2453338.922367$ and $P = 0.23400259$ days.

HJD	Uncertainty	Minima type	Epoch (E)	O–C$_{\text {Primary}}$	O–C$_{\text {Secondary}}$	Facility	Refs.
2453338.922367	0	Primary	0	0.0000	–	TESS	This study
2453339.0381395	0.00002	Secondary	0	–	1.6664	TESS	This study
2453343.60184045	0.00001	Primary	20	0.0142	–	TESS	This study
2453343.48607036	0.00003	Secondary	20	–	0.9736	TESS	This study
2453357.64668321	0.000012	Primary	80	0.0250	–	TESS	This study
2453357.76244677	0.00002	Secondary	80	–	1.5344	TESS	This study
2453360.68891447	0.00012	Primary	93	-0.0065	–	TESS	This study
2453360.80699037	0.00023	Secondary	93	–	0.9464	TESS	This study
2457469.864585	0.00013	Primary	17628	0.0254	–	OAN-SPM	^23^
2457469.98108	0.00157	Secondary	17628	–	1.5345	OAN-SPM	^23^
2457473.84342	0.00524	Primary	17644	-0.0073	–	OAN-SPM	^23^
2457473.95904	0.0061	Secondary	17644	–	0.9933	OAN-SPM	^23^
2460389.5118	0.00017	Primary	30090	0.0025	–	KAO	This study
2460389.3953	0.00018	Secondary	30090	–	1.0215	KAO	This study

## Results and discussion

### Analysis of light curves

The light curve analyses of the three systems were performed using the PyWD2015 software^34^. The program provides a modern graphical user interface (GUI) for the 2015 version of Wilson-Devinney (WD)^35,36^. The adopted parameters, like the gravity-darkening assumed to be g = 0.32^5^, and the bolometric albedo coefficients were fixed as A = 0.5^37^ for both components as appropriate for stars with convective envelopes. The limb-darkening coefficients were derived using the logarithmic law and interpolated from the tabulated values of^38^, which are appropriate for over-contact binary systems. The same coefficients were applied to both components, meaning the linear and non-linear limb-darkening coefficients were set equal: $x_1 = x_2$ and $y_1 = y_2$.

Several methods have been employed in previous studies to calculate the effective temperature of the primary component. Since these approaches often yield varying results, we applied three distinct methods to estimate the effective temperature of the primary component in the studied systems. To improve reliability, we adopted the average of the three estimates as the final value. First, we used the infrared color index (J-H) from the 2MASS catalog and applied Collier’s equation^39^. Second, we utilized the period-temperature relation described by^40^. Third, we retrieved temperature estimates from Gaia^29,41^. Table 5 summarizes the temperature values derived from each method and the calculated mean value.

**Table 5:** average $T_1$ for studies systems.

System	Collir eq	Latković 2021	GAIA DR2	GAIA DR3	Average (T1)
ATO J 255	4474.74	4935.58	4515.63	4860.39	4696.58± 117
CRTS J034	4588.35	4942.73	4485.94	4976.22	4748.31±123
NSVS 266	4566.50	4797.54	4856.34	4569.78	4697.54± 75

**Fig. 3:**
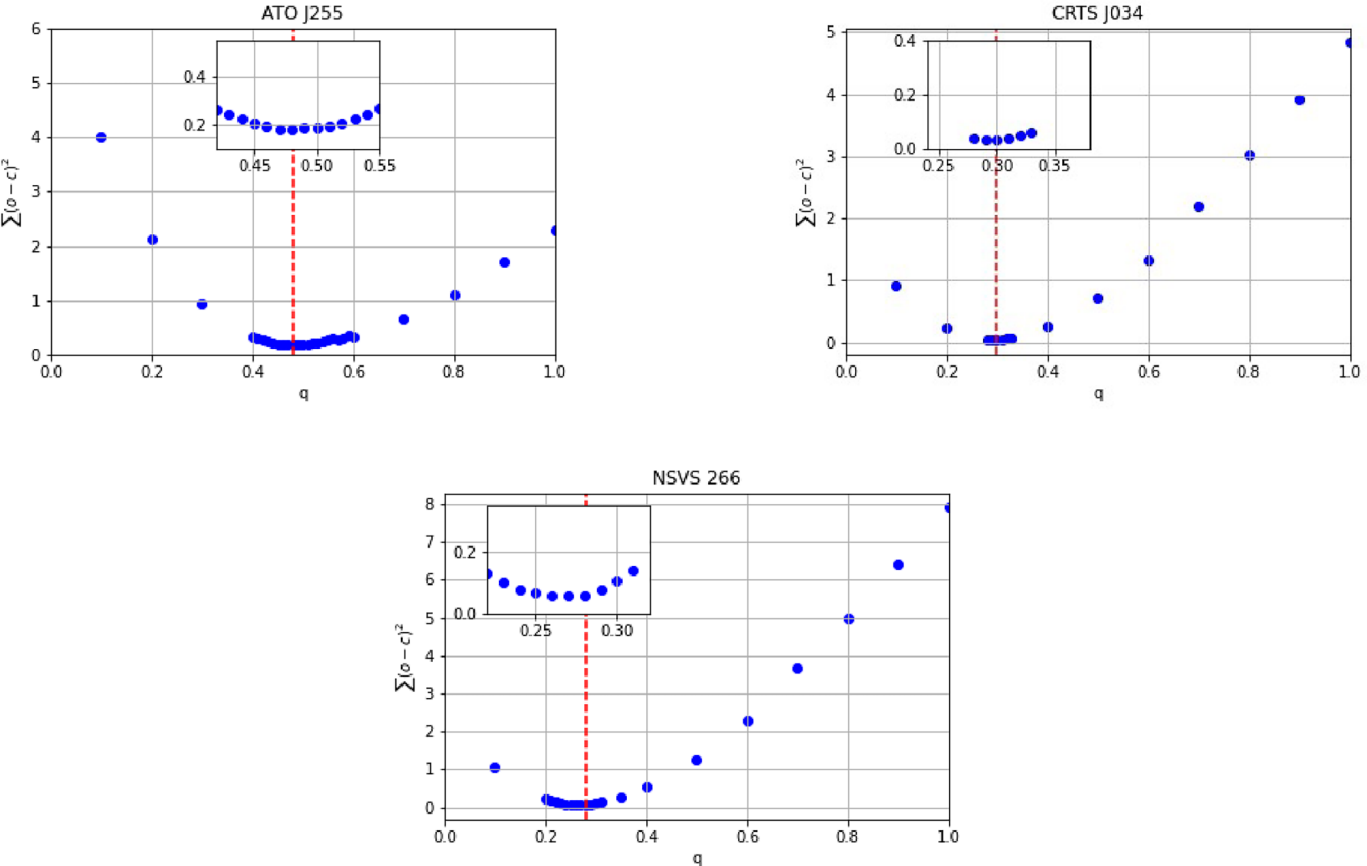
The relation between mass ratio (q) and mean residuals ($\sum (O - C)^2$ ) for the three systems, where the inner subfigure represents zome in over the best q value.

Since there were no available spectroscopic radial velocity measurements for the systems, we adopted the q-search method to determine each system’s mass ratio $q(M_2/M_1)$. This method assigns a specific value to *q* while allowing the other parameters to vary. Figure 3 displays the relationship between the mass ratio (*q*) and the mean residual of each system of input parameters. The best q values for ATO J255, CRTS J034, and NSVS 266 were found to be around 0.48, 0.34, and 0.28, respectively.

The adjustable parameters in this model are the inclination of the orbit (*i*), the potentials ($\Omega _1$, $\Omega _2$), the luminosity of the primary ($L_1$) in each passband, and the temperature of the secondary ($T_2$). Based on the shape of the systems’ light curves, we performed the fits using the overcontact configuration (mode 3), which is suitable for overcontact systems that are not in thermal contact, evidenced by the unequal depths (i.e., temperatures) of the minima.

**Fig. 4:**
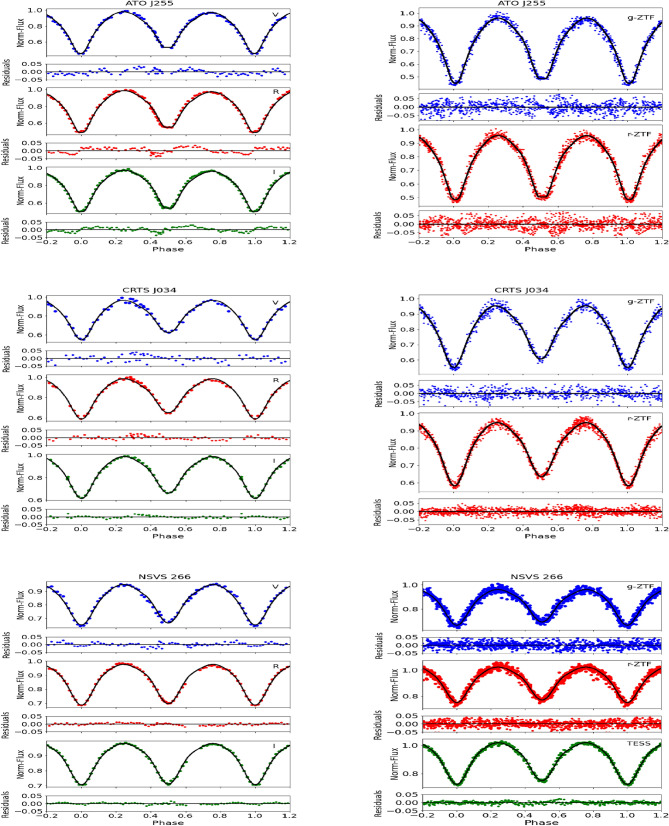
Best theoretical fit (solid line) obtained from light curves analysis together with observed light curves (points) of ATO J255 (top), CRTS J034 (middle), and NSVS 266 (bottom). The left panels show the light curves in the Johnson filters, while the right panels represent the light curves in the ZTF g and r filters. For NSVS 266, an additional light curve obtained using the TESS filter is also included.

Figure 4 shows the best fit between the models and observations for ATO J255, CRTS J034, and NSVS 266. The solutions show an unequal maximum for the ATO J255 system, which may be attributed to the O’Connell effect^22^. This effect is explained by the presence of a cold spot on the primary component of the ATO J255 system (likely caused by magnetic activity in the more massive component). This asymmetry was observed in our data but was not evident in the ZTF archival data. Tables 6,7 and 8 list the solution parameters obtained in the Johnson VRI and ZTF gr filters for ATO J255, CRTS J034, and NSVS 266, respectively. For NSVS 266, results based on TESS data are also included.

**Fig. 5:**
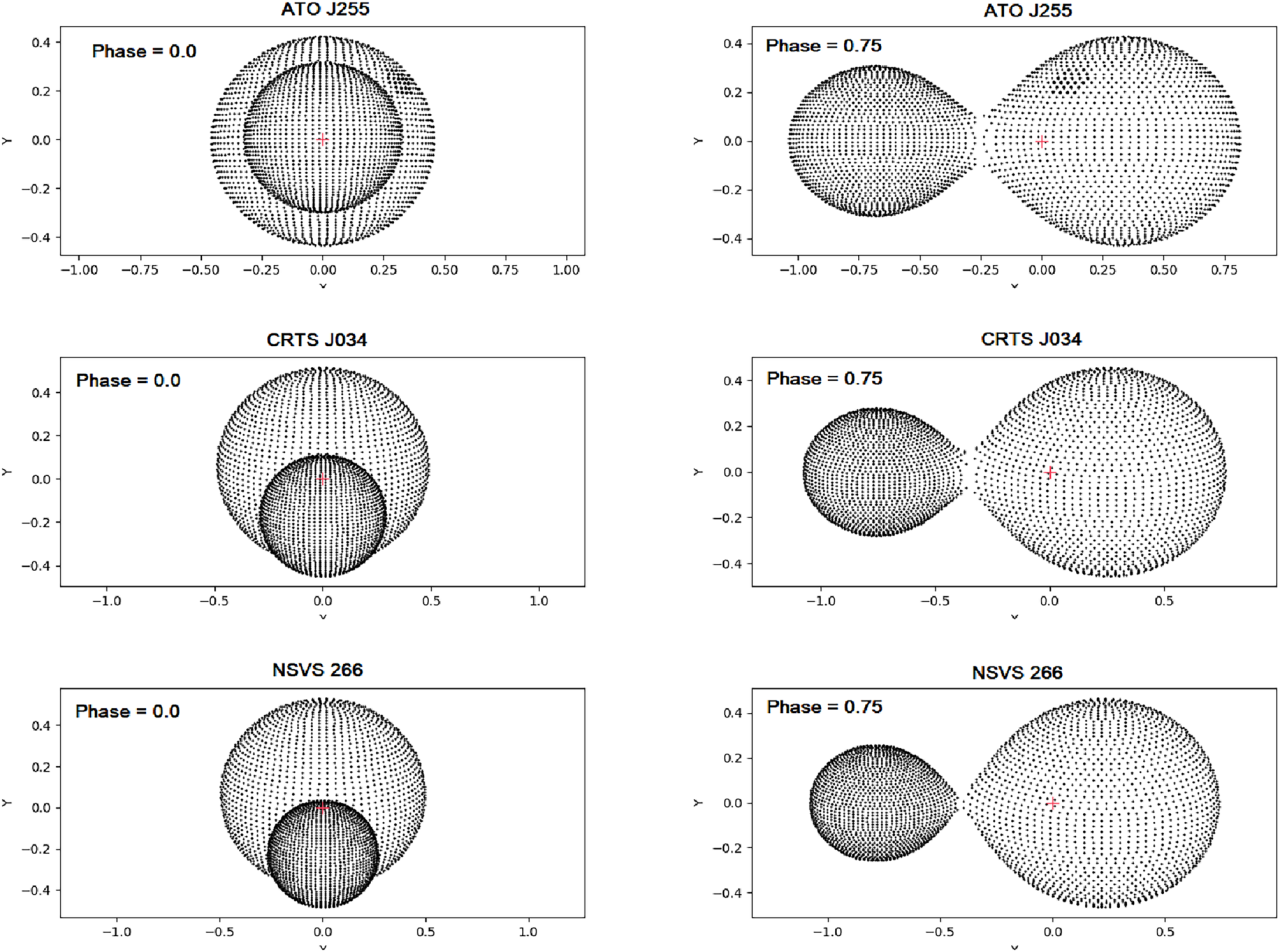
The geometrical configuration of the three systems, based on the fitting parameters obtained in this study.

The accepted W-D parameters were then used to illustrate the three-dimensional configurations of the systems using the PyWD2015 software. Figure 5 shows the over-contact configuration for the three systems with a degree of fill-out as f = 0.204, 0.157, and 0.069 for ATO J255, CRTS J034, and NSVS 266, respectively.

Our solution represents the first analysis of ATO J255 and CRTS J034, while the NSVS 266 system was previously studied by^23^. Comparing our results with those of^23^, we found a difference in the effective temperature of the primary component due to the different methods used. While we calculated $T_1$ as the average of three distinct approaches,^23^ only used the method of^39^. However, the temperature difference between $T_1$ and $T_2$ in both studies remains nearly the same, and there are no significant discrepancies in other parameters such as mass ratio (*q*), potential ($\Omega$), and luminosity of the primary component ($L_1$). while^23^ solved the light curve by incorporating a hot spot on the secondary component. However, the difference between the two maxima in our observations is not significantly large and falls within the observational error range for both ZTF and Kottamia observations. Therefore, we are unable to definitively attribute this asymmetry to the O’Connell effect.

**Table 6:** Light curve solutions of the ATO J255 System.

Parameter	V (5500)	R(7000)	I(9000)	Zg (4746)	Zr (6366)
$T_1$	4696	4696	4696	4696	4696
$T_2$	4621 ± 26	4619±30	4660 ± 28	4680 ± 11	4685 ± 12
I	91 ± 2.7	89.5± 2.4	90.8 ± 1.9	90.7 ± 1.95	89.4 ± 1.58
q	0.47 ± 0.03	0.47± 0.015	0.47±0.013	0.47± 0.010	0.47± 0.006
$\Omega _{1,2}$	2.76 ± 0.04	2.74±0.03	2.77± 0.035	2.78± 0.02	2.76± 0.01
$L_1/(L_1+L_2$)	0.680 ± 0.14	0.665±0.12	0.673±0.02	0.647±0.05	0.647± 0.04
$X_1$	0.806	0.724	0.627	0.842	0.765
$r_1(pole)$	0.4299±0.0028	0.4311±0.0049	0.4245±0.0006	0.4282±0.0019	0.4277±0.0017
$r_1(side)$	0.4600±0.0037	0.4615±0.0061	0.4530±0.0009	0.4578±0.0024	0.4570±0.0021
$r_1(back)$	0.4929±0.0050	0.4947±0.0069	0.4843±0.0016	0.4904±0.0027	0.4893±0.0024
$r_2(pole)$	0.3099±0.0030	0.3116±0.0178	0.3129±0.0015	0.3088±0.0056	0.3120±0.0051
$r_2(side)$	0.3255±0.0036	0.3277±0.0244	0.3289±0.0019	0.3241±0.0071	0.3280±0.0064
$r_2(back)$	0.3678±0.0062	0.3719±0.0845	0.3724 ±0.0037	0.3648±0.0133	0.3717±0.0119
$r_{1 \textrm{mean}}$	0.4602±0.0038	0.4617±0.0059	0.4532±0.0010	0.4580±0.0023	0.4573±0.0020
$r_{2 \text {mean}}$	0.3335±0.0042	0.3361±0.0422	0.3371±0.0023	0.3317±0.0086	0.3363±0.0080
f(%)	20.40	27.52	17.52	16.03	20.48
$\sum (O-C)^2$	0.0089	0.0089	0.0067	0.0127	0.010
Spot parameters (Primary)					
Co-latitude	57.2	57.2	57.2		
Co-longitude	57.2	57.2	57.2		
Radius	6.8	8.5	8.5		
Temperature factor	0.2	0.2	0.2

**Table 7:** Light curve solutions of the CRTS J034 System.

Parameter	V (5500)	R(7000)	I(9000)	Zg (4746)	Zr (6366)
T1	4748	4748	4748	4748	4748
T2	4548±40	4528±30	4528±25	4608±13	4546±10
I	78.48 ± 1.26	77.6 ± 0.86	76.7 ± 0.48	77.08 ± 0.46	76.87 ± 0.29
q	0.34 ± 0.04	0.34 ± 0.03	0.34 ± 0.04	0.34 ± 0.01	0.34 ± 0.01
$\Omega _{1,2}$	2.52 ± 0.02	2.51 ± 0.01	2.52 ± 0.01	2.50 ± 0.02	2.53 ± 0.01
$L_1 / (L_1 + L_2)$	0.775 ± 0.13	0.769 ± 0.08	0.768 ± 0.06	0.769 ± 0.07	0.768 ± 0.04
X1	0.806	0.724	0.627	0.842	0.765
$r_1$ (pole)	0.4530±0.0034	0.4528±0.0022	0.4514±0.0017	0.4526±0.0009	0.4520 ±0.0010
$r_1$ (side)	0.4874±0.0047	0.4870±0.0031	0.4850±0.0021	0.4868±0.0010	0.4858± 0.0013
$r_1$ (back)	0.5162±0.0060	0.5154±0.0039	0.5128±0.0025	0.5155±0.0012	0.5137± 0.0015
$r_2$ (pole)	0.2796±0.0037	0.2783±0.0024	0.2764±0.0071	0.2823±0.0028	0.2737± 0.0033
$r_2$ (side)	0.2925±0.0044	0.2911±0.0029	0.2888±0.0086	0.2957±0.0033	0.2856± 0.0040
$r_2$ (back)	0.3319±0.0078	0.3301±0.0050	0.3262±0.0150	0.3377±0.0060	0.3212± 0.0072
$r_{1 \textrm{mean}}$	0.4848±0.0047	0.4843±0.0030	0.4824±0.0021	0.4842±0.0010	0.4831±0.0012
$r_{2 \textrm{mean}}$	0.3005±0.0053	0.2990±0.0034	0.2963±0.0102	0.3043±0.0040	0.2928±0.0048
$f(\%)$	15.6	18.24	13.94	18.93	12.9
$\sum (O - C)^2$	0.008	0.005	0.003	0.009	0.0069

**Table 8:** Light curve solutions of the NSVS 266 System.

Parameter	V (5500)	R(7000)	I(9000)	Zg (4746)	Zr (6366)	TESS (7697)
T1	4697	4697	4697	4697	4697	4697
T2	4610 ± 29	4625 ± 19	4646 ± 20	4579 ± 11	4574 ± 13	4536 ± 13
I	71.5 ± 0.69	72.38 ± 0.57	71.12 ± 0.34	73.0 ± 0.29	71.0 ± 0.27	71.84± 0.23
q	0.28 ± 0.01	0.28 ± 0.01	0.28 ± 0.01	0.28 ± 0.01	0.28 ± 0.01	0.28 ± 0.013
$\Omega _{1,2}$	2.41 ± 0.01	2.43 ± 0.03	2.42 ± 0.02	2.42 ± 0.01	2.42 ± 0.01	2.42± 0.01
$L_1 / (L_1 + L_2)$	0.780 ± 0.02	0.778 ± 0.02	0.772 ± 0.04	0.795 ± 0.04	0.788 ± 0.03	0.791±0.031
X1	0.806	0.724	0.627	0.842	0.765	0.765
$r_1$ (pole)	0.4631±0.0004	0.4598±0.0036	0.4605±0.0008	0.4616±0.0008	0.4612±0.0007	0.4602±0.0006
$r_1$ (side)	0.4991±0.0006	0.4947±0.0045	0.4956±0.0011	0.4970±0.0010	0.4965±0.0010	0.4952±0.0008
$r_1$ (back)	0.5244±0.0010	0.5188±0.0042	0.5200±0.0014	0.5218±0.0013	0.5212±0.0012	0.5195±0.0010
$r_2$ (pole)	0.2585±0.0017	0.2535±0.0164	0.2551±0.0009	0.2564±0.0008	0.2558±0.0008	0.2549±0.0006
$r_2$ (side)	0.2694±0.0021	0.2636±0.0195	0.2654±0.0010	0.2669±0.0010	0.2663±0.0009	0.2652±0.0007
$r_2$ (back)	0.3038±0.0040	0.2944±0.0332	0.2975±0.0017	0.2998±0.0016	0.2987±0.0015	0.2970±0.0012
$r_{1 \textrm{mean}}$	0.4948±0.0006	0.4904±0.0041	0.4914±0.0011	0.4928±0.0010	0.4923±0.0009	0.4910±0.0008
$r_{2 \textrm{mean}}$	0.2765±0.0026	0.2699±0.0230	0.2720±0.0012	0.2737±0.0011	0.2730±0.0010	0.2717±0.0008
$f(\%)$	6.5	2.3	5.3	5.1	5.1	5.5
$\sum (O - C)^2$	0.005	0.002	0.002	0.008	0.007	0.0036

### Absolute parameters and evolutionary state

We computed the absolute parameters of the three systems following the procedure of^42–44^ as follows: The mass of the primary component ($M_1$) for each system was calculated following^45^.

4 $$\begin{aligned} \log M_1 = 0.755 \log P + 0.416 \end{aligned}$$

where *P* is the period of the system. The secondary ($M_2$) mass is computed using the mass ratio from the light curve solution. Using Kepler’s third law, we computed the semi-major axis a($R_\odot$). Then, the radius (R) of the star is calculated from the relation

5 $$\begin{aligned} R = a*r(mean) \end{aligned}$$

Where $r_{mean}$ outcomes from the light curve solution ($r_{mean} = (r_{back} * r_{side} * r_{pole})^{1/3}$ ). Knowing the effective temperature and radius, we can use the Stefan-Boltzmann law to calculate the luminosity (*L*) of the components as follows:

6 $$\begin{aligned} \frac{L}{L_\odot } = \left( \frac{R}{R_\odot } \right) ^2 \left( \frac{T_{\text {eff}}}{T_{\text {eff} \odot }} \right) ^4 \end{aligned}$$

The bolometric magnitude ($M_{bol}$) of the star is calculated from:

7 $$\begin{aligned} M_{\text {bol}} - M_{\text {bol} \odot } = -2.5 \log \left( \frac{L}{L_\odot } \right) \end{aligned}$$

Where the bolometric magnitude of the sun ($M_{bol_{\odot }}$) is 4.73^46^. The absolute magnitude ($M_V$) calculated using the following relation

8 $$\begin{aligned} M_V = M_{bol} - BC \end{aligned}$$

Where the bolometric correction (BC) is extracted from Fowler’s tables^47^. The absolute parameters of the three systems are listed in Table 9. The errors in absolute parameters are calculated based on the uncertainties in the input parameters using the following equation:

9 $$\begin{aligned} \sigma _f = \sqrt{\left( \frac{\partial f}{\partial x_1} \sigma _{x_1} \right) ^2 + \left( \frac{\partial f}{\partial x_2} \sigma _{x_2} \right) ^2 } \end{aligned}$$

Where f is the derived parameter, xi is the input parameter, $\sigma x$ are the uncertainties in $x_i$, and $\delta f/\delta x$are the partial derivatives.

**Table 9:** Absolute parameter of the three systems ATO J255, CRTS J034, and NSVS 266.

System	Component	BC	Mbol	Mv	T ($T_\odot$)	M ($M_\odot$)	R ($R_\odot$)	L ($L_\odot$)	P (days)	log *J*_0_(cgs)
ATO J255	Primary	-0.471	6.11	6.59	0.81	0.91±0.09	0.79±0.03	0.27±0.03	0.251745	51.44
	Secondary	-0.517	6.90	7.41	0.79	0.43±0.04	0.57±0.02	0.135±0.01		
CRTS J034	Primary	-0.437	6.01	6.45	0.82	0.92±0.09	0.81±0.04	0.30±0.04	0.252665	51.32
	Secondary	-0.564	7.25	7.81	0.78	0.31±0.04	0.50±0.03	0.09±0.01		
NSVS 266	Primary	-0.471	6.20	6.67	0.81	0.87±0.08	0.76±0.04	0.25±0.03	0.2340026	51.19
	Secondary	-0.528	7.55	8.07	0.79	0.24±0.03	0.42±0.02	0.07±0.01		

**Fig. 6:**
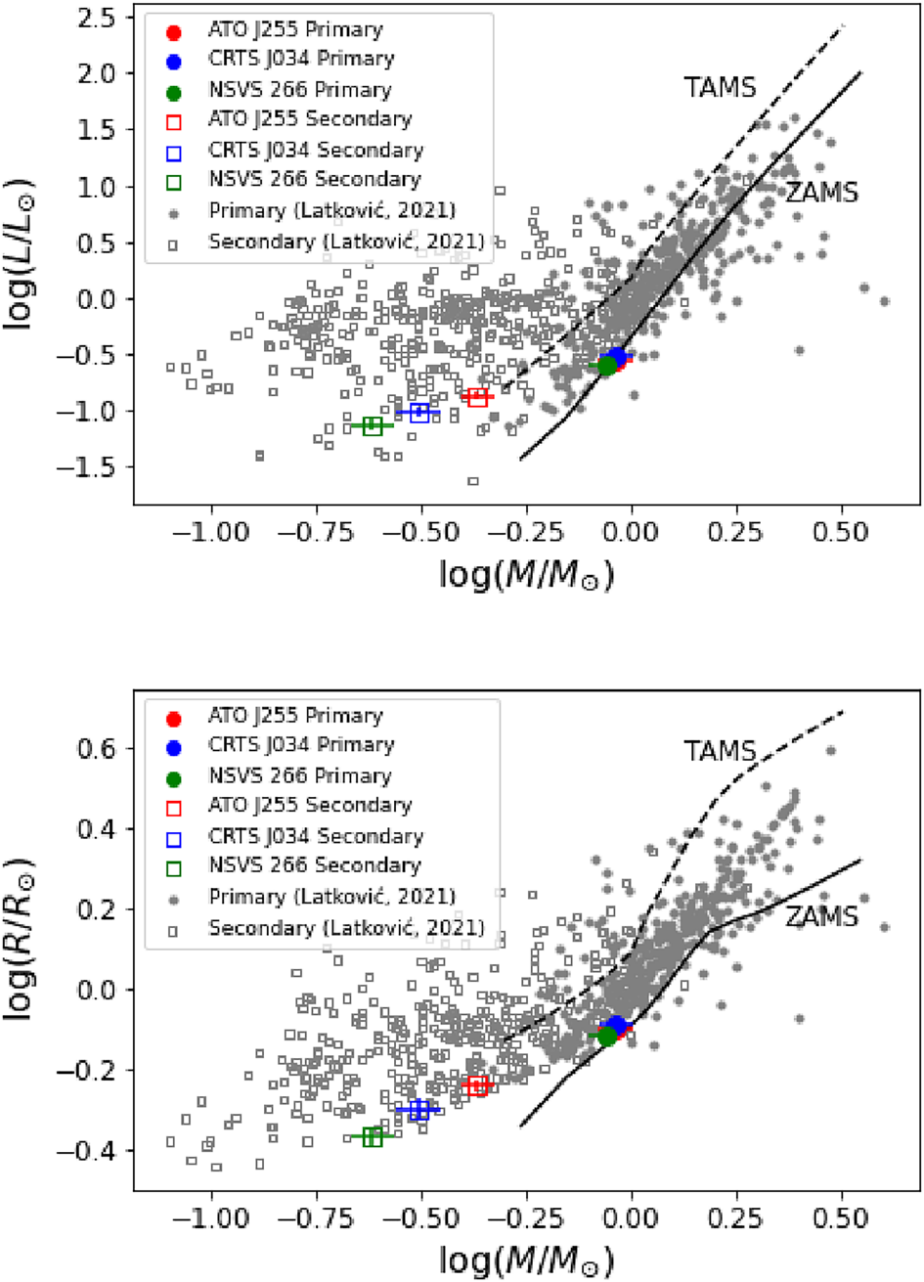
Locations of the components of ATO J255 (red symbols), CRTS J034 (blue symbols), and NSVS 266 (green symbols), by comparison with the contact binary sample from Latkovi? (2021) on mass-luminosity (upper) and mass-radius (middle) relation (^48^). The circles represent the primary components, while the squares represent the secondary components.

**Fig. 7:**
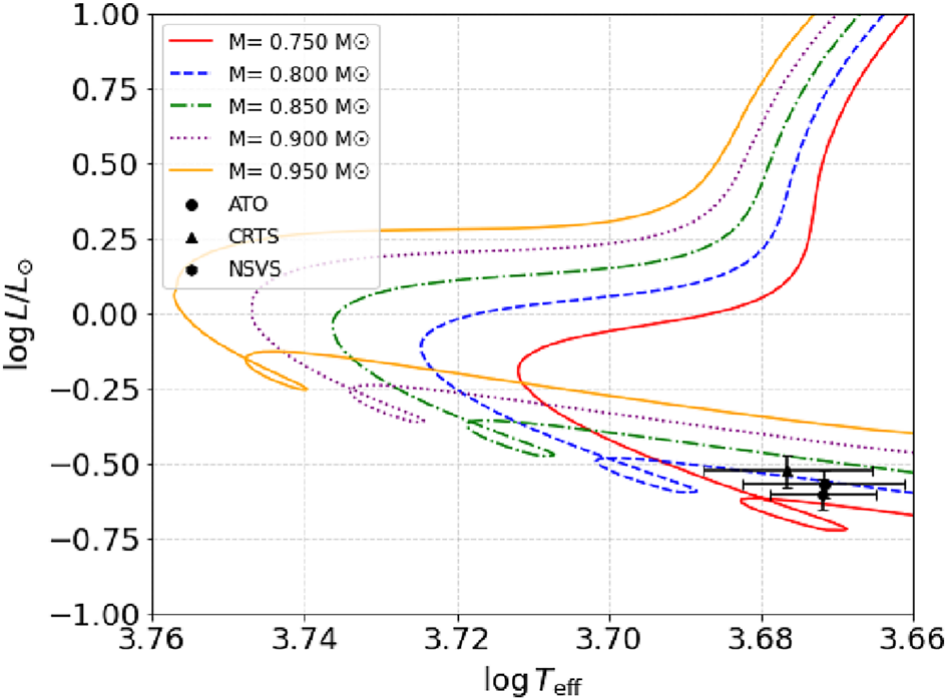
Positions the primary component of each system on $log T_{eff} - log L/L_\odot$ plane. Evolutionary tracks and isochrones are from^49^. Each track corresponds to the mass value of solar units.

The positions of the individual components of the system are shown in two diagrams: the Mass-Luminosity (M-L, upper panel) and Mass-Radius (M-R, bottom panel) relations for the Zero Age Main Sequence (ZAMS) and the Terminal Age Main Sequence (TAMS) based on^48^. The circles denote the primary components, while the squares represent the secondary components. These diagrams in Fig. 6 are used to assess the system’s evolutionary stage, where the primary components of the three systems are located over the main sequence while the secondary components of the three systems are out of the main sequence, which indicates that they are in the evolution stage. To further investigate the evolution of our studied systems, we compared our results with contact binary systems listed in the contact binary Catalog compiled by^40^ (https://wumacat.aob.rs/Stars). This catalog provides a comprehensive dataset of well-characterized contact binaries, allowing us to place our studied systems within evolutionary tracks; the contact binary sample from^40^ was plotted in Fig. 6 as black circles and squares for primary and secondary components, respectively. Using pre-main sequence evolutionary tracks from Bressan et al. (2012), we located primary components over the $log T_{eff}-log L/L_\odot$ plane (Fig. 7) to verify that they are main-sequence stars. The results show that all three primary components align with the pre-main sequence track

**Fig. 8:**
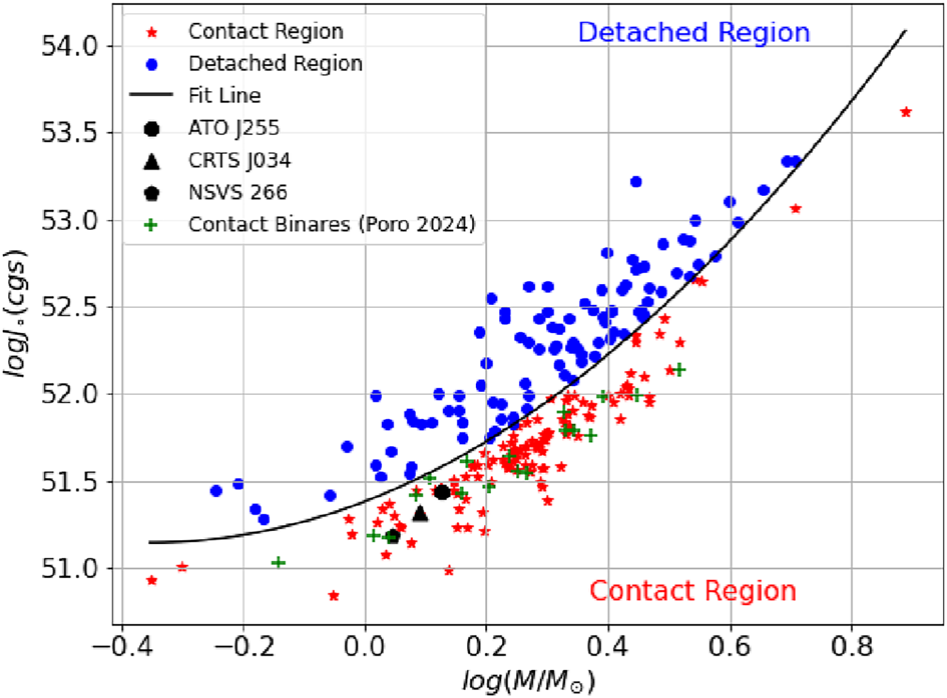
The locations of ATO J255, CRTS J034, and NSVS 266 on the log Jo-log M diagram. The other symbols indicate the contact and detached binaries from^50^. Additionally, known contact binaries compiled by^51^ were also included.

The orbital angular momentum (OAM, Jo) and total mass (M) of the system are fundamental physical quantities that determine the orbital size (a) and period (P). The OAM and mass loss are the physical parameters that control the magnitude and direction of dynamical evolution, so the log Jo-log M diagram is an obvious choice for studying the dynamical evolution of binary orbits. We calculated the orbital angular momentum of the three systems using the following equation^50^.

10 $$\begin{aligned} J_0 = \frac{q}{(1+q)^2} \root 3 \of {\frac{G^2 M^5 P}{2\pi }} \end{aligned}$$

where *Jo* is the orbital angular momentum in $g cm^{2} s^{-1}$, *q* is the mass ratio, *M* is the total mass of the binary, *P* is the orbital period, and *G* is the gravitational constant. The three binaries have *logJo* values 51.4660 (ATO J255), 51.3226 (CRTS J034), and 51.5414 (NSVS 266) in the *cgs* system. In Fig. 8, we plot the orbital angular momentum against the total mass distribution. By placing our studied systems on this diagram alongside the known contact binary systems of^51^, we found that the three systems follow the same trend as the known contact binaries. They lie within the contact region, below the quadratic boundary line defined by^50^, as expected for contact binary systems.

**Fig. 9:**
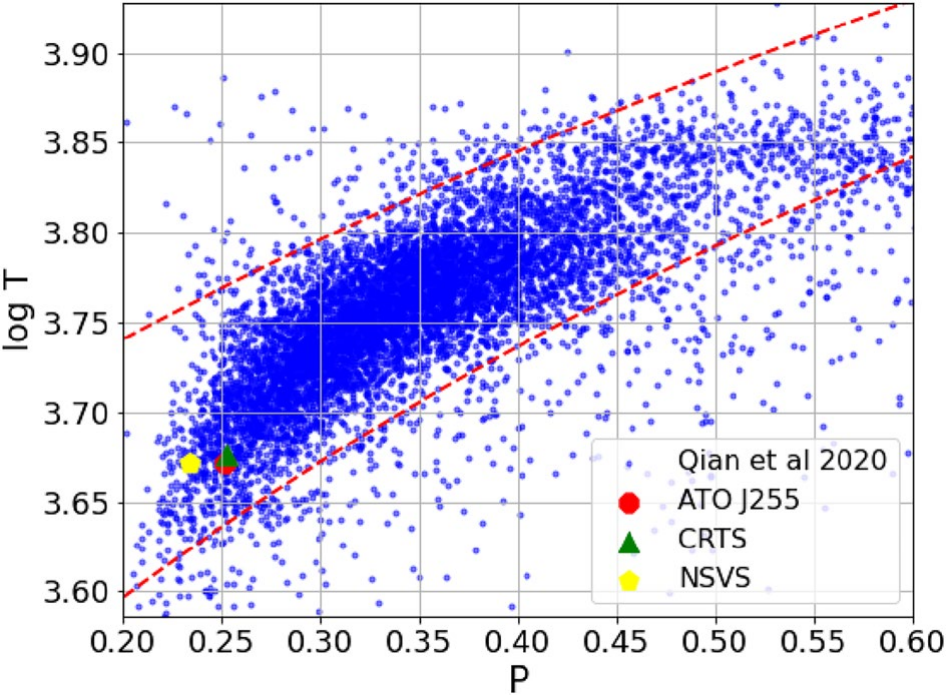
The p-log T relation for contact binaries observed by LAMOST. The parameters of our three systems are overplotted on the plot.

The period-color (or temperature) relationship (Eggen 1967;^37^) is a well-established correlation for contact binaries. All EW-type eclipsing binaries listed in the variable star index (VSX) exhibit a period distribution consistent with the relative period distribution of EWs identified through LAMOST low-resolution spectroscopy (LRS). This similarity suggests that LAMOST contact binaries can be effectively used to study the overall properties of EWs. Given the correlation between orbital period and effective temperature observed in 8,510 contact binaries by LAMOST, the majority of EWs fall within the normal EW limits defined by^52^. Figure 9 presents the period-temperature distribution for contact binaries, with the three studied systems plotted on the $P-log T$ diagram. Their positions lie between the two boundary lines (dashed red lines), indicating that they are likely normal contact binaries.

## Conclusions

In the present study, we report new light curves for three eclipsing binary systems, ATO J255, CRTS J034, and NSVS 266, based on recent observations conducted at the Kottamia Astronomical Observatory, as well as archival data obtained from the Zwicky Transient Facility (ZTF) and the *Transiting Exoplanet Survey Satellite* (TESS). Through detailed light curve modelling, we have derived the physical parameters of the three systems. New times of minima for all three systems have been calculated. In particular, for NSVS 266, we performed an O-C (Observed-Calculated) analysis to investigate period variations. The results indicate a decreasing orbital period, with a rate of $\textrm{d}P/\textrm{d}t \approx 1.85 \times 10^{-10}$ days yr$\phantom{0}^{-1}$.

Based on the systems’ mass ratios and surface temperatures, we classified all three as A-subtype W UMa contact binaries. Contact binaries are typically categorized by their fill-out factor into three groups: *deep contact* ($f \ge 50\%$), *medium contact* ($25\% \le f < 50\%$), and *shallow contact* ($f < 25\%$) systems^53^. According to this classification, ATO J255, CRTS J034, and NSVS 266 are all shallow contact systems, with fill-out factors of 0.204, 0.157, and 0.069, respectively. The light curve of ATO J255 displays an O’Connell effect, which we attribute to the presence of a cool spot on the primary component. This feature provides insight into magnetic activity and starspot evolution in contact binary systems. However,^54^ reported that the accuracy of the mass ratio (*q*) becomes less reliable as the eclipses change from total to partial. This suggests that the value of *q* obtained for ATO J255 is likely more accurate than those for CRTS J034 and NSVS 266. Nevertheless, we think that high-quality photometric observations can still lead to reasonably accurate determinations of *q*, even in systems with partial eclipses.

Using the derived mass ratios and a set of empirical relations, we estimated the absolute physical parameters of the three systems. These parameters are expected to be more reliable for ATO J255, while CRTS J034 and NSVS 266 would benefit from additional spectroscopic observations to improve parameter accuracy. A better understanding of the physical and evolutionary states of these systems requires precise measurements of absolute parameters such as mass, radius, and luminosity. Our photometric estimates provide an initial approximation of their evolutionary stages. The positions of the components on mass-luminosity and mass-radius diagrams suggest that the primary components remain on the main sequence, whereas the secondary components exhibit signs of evolution. To assess the reliability of our results, we compared the absolute parameters of our systems with a well-characterised sample of contact binaries from^40^. The components of our systems follow the same general trends as those in the comparison sample, supporting the validity of our derived parameters. Moreover, the calculated angular momentum values further confirm the classification of these systems as contact binaries.

The current study contributes to future observational and theoretical studies, particularly in binary star stability and mass transfer dynamics. Future spectroscopic studies are recommended to refine the estimates of mass, temperature, and radial velocity curves, thereby enhancing our understanding of the internal structure and evolutionary status of these systems.

## Data Availability

The datasets used and/or analysed during the current study are available from the corresponding author upon reasonable request The data that supports the findings of this study is available at ZTF Public Releases (https://irsa.ipac.caltech.edu/Missions/ztf.html).
